# About the urachus and its pathology A clinical case of urachus tumor

**Published:** 2009-04-25

**Authors:** Bratu Ovidiu, Madan Victor, Ilie Cristian, Rusu Florin, Ghilic Ciprian, Farcas Catalin, Mischianu Dan

**Affiliations:** * Urology Clinic, “Dr. Carol Davila” Clinical Central Military Emergency Hospital, Bucharest, Romania

## Abstract

Urachus diseases represent a relatively rare kind of affliction in child or adult abdominal or urological surgical pathology.

The preservation of the urachus lumen leads to rare afflictions, noticed mostly after birth or when they become clinically manifest by various complications.

More often than not, these pathological entities require surgical interventions (if the urachal lumen does not close by itself), consisting of partial or total excision of the urachus.

Tumor pathology is mostly malign, to a large extent represented by urachus adenocarcinoma. Its initial symptomatology is scarce and confusing.

Its treatment is mainly by surgery and consists of the surgical excision of the urachal ligament, of the umbilicus, of a part of the front abdominal wall and partial or total cystectomy, as necessary.

The prognosis is unfavourable, since urachal adenocarcinoma is deemed to be a particularly aggressive tumor, strongly influenced by the status of the excision edges, that is by the radicalness of the surgical intervention. Irrespective of the latter, an adjuvant oncological treatment is to be prescribed, mainly systemic cytostatic therapy.

Urachus adenocarcinoma is rarely encountered and very often diagnosed in late metastatic stages, when the only solution is at most paleative surgery.

Urachus diseases represent a relatively rare kind of affection in child or adult abdominal or urological surgical pathology. The largest part of the pathology of this so-called “organ” is considered to be congenital malformation. 

This study is meant to mention the main urachus diseases, with emphasis on malign pathology, since it presents a number of characteristics we are to list in due time. Likewise, we will describe an unusual clinical case, diagnosed and treated in our clinic.

We will begin by mentioning some anatomic data regarding this organ. The urachus is a tubular organ, mostly obstructed, and represents a vestige of the allantois, which links the umbilicus to the urinary bladder dome during intra-uterine life. The urachus lumen begins to close as early as the 4-5 months of embryonic life, from the bladder to the umbilicus, as the bladder descends into the pelvis. Anatomically speaking, it is 5-10 cm long, with a diameter of 3-7 mm and represents the mesial line of the umbilical-prevesical fascia. Laterally of the urachus, starting from the umbilicus, the vestiges of the umbilical arteries begin and mark the supravesicular fossetae. Both the urachus and the obliterated umbilical arteries are important landmarks in abdominal surgery, especially in the case of inguinal hernias.

The preservation of the urachus lumen leads to unusual afflictions, noticed mostly after birth or when they are clinically manifested by various complications. Specialized literature lists them in the order of their frequency, as it follows: external urachal sinus, internal urachal sinus or diverticulum, urachal cyst and permeable urachus (the latter taking the form of a genuine vesicular-umbilical fistula).

If the anomaly is not obvious, the diagnosis will be usually applied on the appearance of complications, as in the case of external urachal sinus, which may lead to evacuation of the (often purulent) content at the level of the umbilicus or to urinary complications, with pyuria and recurrent urinary infections in case of an over infected urachal diverticulum. 

More often, these pathological entities require surgical intervention (if the urachal lumen does not close by itself), consisting of partial or total excision of the urachus.

Tumor pathology is mostly malignant, largely represented by urachus adenocarcinoma. The epithelium that lines the urachus lumen is cuboid and becomes transitional towards the vesicular dome. The urachus adenocarcinoma is supposed to be caused by the malign degenerescence of some urachal cyst epithelium, something that has not been confirmed so far. Usually, these tumors show a clear-cut delimitation between tumor epithelium and urinary bladder epithelium. There are cases of sarcomas and transitional or squamous cell carcinomas, which also contain adenocarcinoma elements in their histopathological pattern.

Urachus adenocarcinoma is a rare type of tumor, with an incidence of about 1/1,500 vesicular tumors and representing about 0.7% of the overall number of tumors.

Its clinical manifestations may be abdominal (especially periumbilical) pain, liquid leaks (serous, purulent, blood-streaked) from the level of the umbilicus, possible dysuria, haematuria, intermittent pyuria or urinary infections (apparent in positive urine cultures) due to the evacuation of the infected tumor content into the urinary vesicle. Abdominal touch may reveal fixed or mobile tumor formations, usually subumbilical.

The prevailing imagistic investigation in diagnosing urachal adenocarcinoma is computerized tomography, which reveals the tumor properly, the distant invasion, the invasion of the vesicular dome, as well as possible metastatic adenopathies. Other investigation methods may be intravenous urography, which shows the dispersion of the contrast substance at the level of the vesicle dome, and nuclear magnetic resonance imaging, which does not provide any new data in relation to the CT.

If the patient’s symptomatology is, for instance, only intermittent macroscopic haematuria, the diagnosis of urachal adenocarcinoma may be tackled starting from a cystoscopic test, which is part of the standard protocol for diagnosing haematuria.

Immunologically speaking, since it has a typical intestinal pattern, urachal adenocarcinoma may determine increased values of the carcinoembryonary antigen, CA 19-9 or CA 125. These are not specific markers and they cannot be used as a diagnostic test; however, they may be useful in estimating the scope of a surgical intervention or of distance post-operative observation.

Several systems of delimiting the stages of urachal adenocarcinoma have been suggested, among which the most widely used is that established by Sheldon (1994), as it follows:

I. No invasion beyond the urachal mucosa

II. Limited invasion to the urachus

IIIA. Local extension in the bladder

IIIB. Local invasion in the abdominal wall

IIIC. Local invasion in the peritoneum

IIID. Local invasion in an organ other than the bladder

IVA. Metastasis in the regional lymphatic ganglions

IVB. Distant metastasis

The treatment of urachus adenocarcinoma is done mainly by surgery and consists of the surgical excision of the urachal ligament, of the umbilicus, of a part of the front abdominal wall and partial or total cystectomy, if necessary.

The prognosis is unfavorable, since urachal adenocarcinoma is deemed to be a particularly aggressive tumor, strongly influenced by the status of the excision edges, that is, by the radicalness of the surgical intervention. Irrespective of the latter, an adjuvant oncological treatment is to be prescribed, mainly systemic cytostatic therapy.

We will present below a particular case of urachal adenocarcinoma of a 50-year old male patient, hospitalized in our clinic for gas and faeces in his urine, as established by anamnesis. During his stay in the hospital, he developed intestinal transit troubles, as well as a suboclusive syndrome, which determined us to intervene before he was submitted to a computerized tomography of the abdomen.

Laboratory tests revealed nothing significant. Nothing wrong in the urine summary or in the urine culture. Clinically, between the umbilicus and the pubes, touch revealed a firm mobile tumor formation of about 10/10cm. The tumor markers showed three times higher values of CEA and CA 19-9, while CA 125 and alpha-fetoprotein were within normal values.

Suspecting a digestive tumor with influence on the bladder, respectively a possible sigmoid-vesicular fistula, we started the imagistic investigation.

The urinary system echography was within normal limits but the abdominal one revealed the presence of the 10/10 cm tumor formation, well vascularized according to the Doppler test, as well as an important amount of liquid in the abdominal cavity.

We practiced cystoscopy under rachianaesthesia, thus revealing the presence of a vesical tumoral formation at the level of the vesicle dome, with macroscopic characteristics distinct from an ordinary transitional carcinoma.

**Fig. 1:**
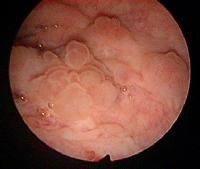
Cystoscopic test – tumor fringes at the level of the vesicle dome

Intravenous urography showed the dispersion of the contrast substance at the level of the vesicular dome.

**Fig. 2:**
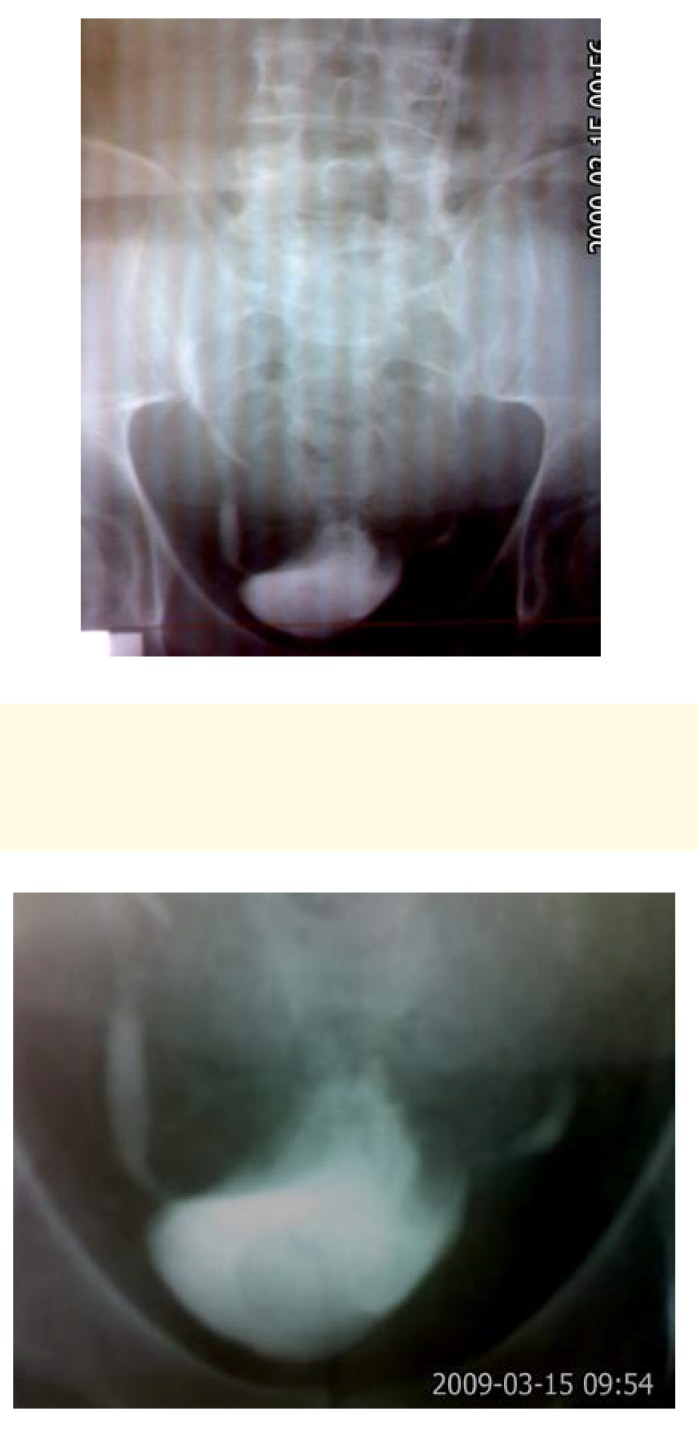
Urographic sequence showing the dispersion of the contrast substance at the level of the vesicle dome

We also practiced irigography, because of the suspicion of enterovesicular fistula.

**Fig. 3:**
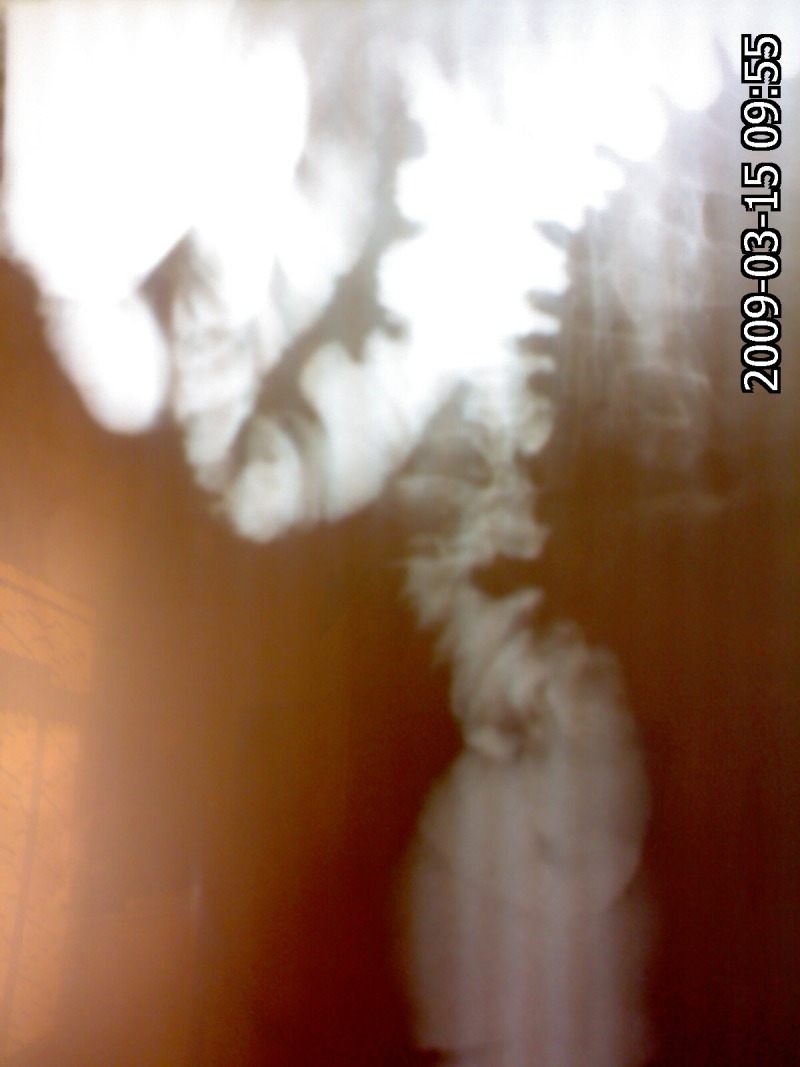
Irigographic image showing the irregular form of the sigmoid colon haustrae

We recurred to surgery, namely pubic-xyphoidian incision, and during the intervention, we discovered a tumor mass extending from the level of the umbilicus to that of the vesicular dome, to which it adhered and infiltrated with tumor cells. We evacuated about 2 liters of neoplasic ascitis liquid and noticed the presence of peritoneal carcinomatosis, with numerous tumor disseminations at the level of intestinal ansae and their mesos, announcing a future occlusive syndrome. The great epiploon was completely infiltrated with tumor cells; the sigmoid colon was very adherent to the tumor formation in the Douglas cul-de-sac. Periaortic and pericaval adenopathies were also present, from the level of the diaphragm to the bifurcation of the great blood vessels.

**Fig. 4:**
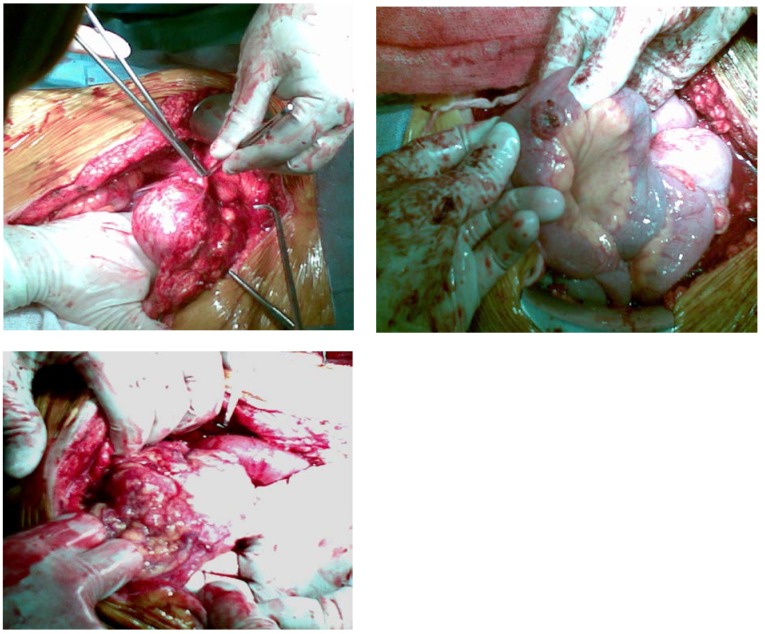
Intra-surgery images – urachal tumor formation extending towards the vesicle dome, adherent to the front abdominal wall, which it infiltrates with tumor cells; multiple intestinal metastasis, suggesting peritoneal carcinomatosis; total invasion of the great epiploon

Taking into account all these aspects, a radical surgical intervention was out of question. Therefore, we performed the resection of the primary tumor formation, together with the umbilicus and the infiltrated abdominal wall, as well as partial cystectomy and palliative omentectomy.

**Fig. 5:**
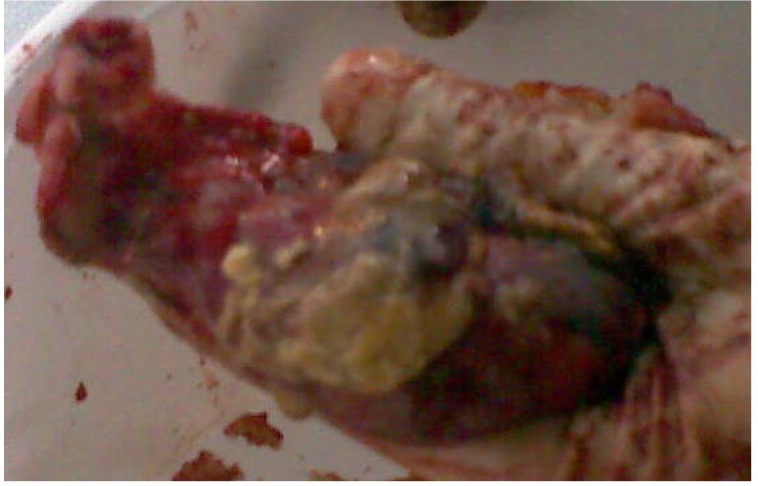
Post-surgery image of the main resected piece. One may notice the central necrosis of the tumor, as well as the vesicle dome infiltrated by tumor cells

The post-surgery evolution was favorable. The tumor markers basically retained the same values as before surgery, which was actually expected. The histopathological test revealed a mixed colloid and intestinal urachal adenocarcinoma, with invasion of the muscular tunic of the bladder and with extensive necrosis areas. 

Faced with a case of such overwhelming surgical radicalness we can only draw a few conclusions.

Urachal adenocarcinoma is an aggressive tumor pathology. It is rarely encountered and very often diagnosed in late metastatic stages, when the only solution is at most palliative surgery. Its initial symptomatology is scarce and confusing, as in the case we have described. The prognosis will obviously be pessimistic, even in the situation of aggressive polychemiotherapy. Survival in such cases is estimated to be at most 2 years from the moment of diagnosis. 

Consequently, it might be useful to consider that all tumors located in the vesicular dome area are of urachal origin, until proven otherwise. The treatment must consist of extremely aggressive surgery, with intraoperative delimitation of the tumor-free edges, by multiple randomized biopsies of the surrounding areas after resection. Certainly, the invasion of the peritoneum, lymphatic ganglions, and other organs, basically makes it a lost battle from the start…
